# Comparative Chloroplast Genomics of *Corydalis* Species (Papaveraceae): Evolutionary Perspectives on Their Unusual Large Scale Rearrangements

**DOI:** 10.3389/fpls.2020.600354

**Published:** 2021-01-27

**Authors:** Xiaodong Xu, Dong Wang

**Affiliations:** ^1^School of Life Sciences, Central China Normal University, Key Laboratory for Geographical Process Analysis and Simulation, Wuhan, China; ^2^Bio-Resources Key Laboratory of Shaanxi Province, Shaanxi University of Technology, Hanzhong, China

**Keywords:** *Corydalis*, plastome, rearrangement, relocation, inversion, IR expansion, pseudogenization

## Abstract

The chloroplast genome (plastome) of angiosperms (particularly photosynthetic members) is generally highly conserved, although structural rearrangements have been reported in a few lineages. In this study, we revealed *Corydalis* to be another unusual lineage with extensive large-scale plastome rearrangements. In the four newly sequenced *Corydalis* plastomes that represent all the three subgenera of *Corydalis*, we detected (1) two independent relocations of the same five genes (*trnV-UAC*-*rbcL*) from the typically posterior part of the large single-copy (LSC) region to the front, downstream of either the *atpH* gene in *Corydalis saxicola* or the *trnK-UUU* gene in both *Corydalis davidii* and *Corydalis hsiaowutaishanensis*; (2) relocation of the *rps16* gene from the LSC region to the inverted repeat (IR) region in *Corydalis adunca*; (3) uniform inversion of an 11–14 kb segment (*ndhB*-*trnR-ACG*) in the IR region of all the four Corydalis species (the same below); (4) expansions (>10 kb) of IR into the small single-copy (SSC) region and corresponding contractions of SSC region; and (5) extensive pseudogenizations or losses of 13 genes (*accD*, *clpP*, and 11 *ndh* genes). In addition, we also found that the four *Corydalis* plastomes exhibited elevated GC content in both gene and intergenic regions and high number of dispersed repeats. Phylogenomic analyses generated a well-supported topology that was consistent with the result of previous studies based on a few DNA markers but contradicted with the morphological character-based taxonomy to some extent. This study provided insights into the evolution of plastomes throughout the three *Corydalis* subgenera and will be of value for further study on taxonomy, phylogeny, and evolution of *Corydalis*.

## Introduction

The chloroplast genome (plastome) of angiosperms (particularly photosynthetic members) is generally highly conserved in terms of structural organization, gene content, and gene arrangement (Palmer, 1985; Wicke et al., 2011; Ruhlman and Jansen, 2014; Mower and Vickrey, 2018). Plastome rearrangements, if they occurred, tend to be relatively minor. Large-scale rearrangement is rare, but it occasionally exists in a few lineages, such as Campanulaceae (Knox et al., 1993; Cosner et al., 2004; Knox, 2014; Knox and Li, 2017; Uribe-Convers et al., 2017, etc.), Geraniaceae (Palmer et al., 1987; Chumley et al., 2006; Guisinger et al., 2011; Weng et al., 2014, 2017; Röschenbleck et al., 2016, etc.), Fabaceae (Kolodner and Tewari, 1979; Palmer and Thompson, 1981; Lavin et al., 1990; Doyle et al., 1996; Cai et al., 2008; Martin et al., 2014; Schwarz et al., 2015; Wang et al., 2017, etc.), Oleaceae (Lee et al., 2007), Asteraceae (Jansen and Palmer, 1987; Kim et al., 2005; Sablok et al., 2019, etc.), Plantaginaceae (Zhu et al., 2016; Kwon et al., 2019; Asaf et al., 2020, etc.), and Poaceae (Palmer and Thompson, 1982; Doyle et al., 1992; Michelangeli et al., 2003; Burke et al., 2016; Liu et al., 2020, etc.). Given that the structure of the two already known plastomes from the genus *Corydalis* was highly variable (Wu et al., 2020; Xu and Wang, 2020), this genus is expected to represent an overlooked lineage with extensive large-scale plastome rearrangements. However, understanding of the pattern, origin, and evolution of plastome rearrangements within *Corydalis* is presently limited by the paucity of plastome sequences.

*Corydalis* (∼465 species) is the largest genus within Papaveraceae and one of the largest genera in Chinese flora (Wu et al., 1999; Zhang et al., 2008). *Corydalis* species, with a wide distribution in north temperate regions, are particularly diverse in the Hengduan Mountains and Qinghai–Tibet Plateau and adjacent areas (Wu et al., 1999; Zhang et al., 2008). Along with the uplift of the Hengduan Mountains and Qinghai–Tibet Plateau, *Corydalis* species have experienced intensive and rapid differentiation, and their plastomes must have also undergone a series of genetic shift to adapt to the drastically changed environment. *Corydalis* may represent an appropriate group to explore how the plastome content and structure have varied in a fine scale in the evolution history, when the unusual plastome rearrangements have originated, and why those changes have happened. In addition, some *Corydalis* plants have the potential to be exploited as medicine for their anti-hepatitis, antitumor, cardiovascular disease treatment, and pain-releasing effects (Luo et al., 1984; Editorial Board of Chinese Tibetan Medicine, 1996; Chinese Pharmacopoeia Commission, 2015; Zhang B. et al., 2016, etc.). However, the complexity of their morphological characters has greatly challenged our understanding of their taxonomy, ecology, evolution, and utilization. Based on morphological characters and/or a few molecular markers, previous studies (Fedde, 1936; Lidén, 1986; Lidén et al., 1995, 1997; Wu et al., 1996, 1999; Wang, 2006; Zhang et al., 2008; Zhang Z.X. et al., 2016; Jiang et al., 2018; Ren et al., 2018; Xu and Wang, 2018, etc.) have made positive contributions to the taxonomy and systematics of *Corydalis*. Until now, a robust backbone phylogeny of this genus, which is instructive for taxonomy and systematics, is still not completed due to the lack of enough genetic resources.

Complete plastome data have been applied to resolve long-standing controversies at different taxonomic levels (e.g., Jansen et al., 2007; Moore et al., 2007, 2010; Ma et al., 2014; Barrett et al., 2016; Zhai et al., 2019). The highly divergent regions of the plastome can be identified as DNA barcodes for future phylogenetic and population genetic analyses. Meanwhile, the plastome rearrangements can also be useful phylogenetic markers, because they typically lack homoplasy and are easily identified (Jansen and Palmer, 1987; Downie and Palmer, 1992; Doyle et al., 1996; Cosner et al., 2004). So far, only two *Corydalis* plastomes were formally, but simply, reported (Wu et al., 2020; Xu and Wang, 2020).

In the present study, we newly sequenced the plastomes of four *Corydalis* species representing all the three subgenera of *Corydalis* and conducted detailed comparative genomic analyses of them with the two previously reported *Corydalis* plastomes and the rest of the Ranunculales plastomes as well. Specifically, we aimed to (1) determine the plastome structure, gene content, and gene arrangement of all the three subgenera of *Corydalis*; (2) explore the pattern, origin, evolution, and phylogenetic utility of plastome rearrangements in *Corydalis*; (3) evaluate the effectiveness of complete plastome in phylogenetic analyses; and (4) screen the highly informative plastome DNA regions for future Sanger-based studies.

## Materials and Methods

### Taxon Sampling, DNA Extraction, Library Construction, and Sequencing

Samples of four *Corydalis* species (i.e., *Corydalis adunca* Maxim., *Corydalis saxicola* Bunting, *Corydalis hsiaowutaishanensis* T. P. Wang, and *Corydalis davidii* Franch.), representing all the three subgenera of *Corydalis*, were collected from their wild population (Table 1). Voucher specimens were deposited in the herbarium of the Central China Normal University (CCNU), Wuhan, China. Fresh leaves were dried in the wild using silica gel and preserved at −20°C until DNA extraction. Plants were identified following the treatment of *Corydalis* in the *Flora of China* (Zhang et al., 2008). DNA extraction, library preparation, and sequencing were conducted at Novogene (Tianjin, China). Total DNA was isolated using the modified cetyl trimethylammonium bromide (CTAB) method (Doyle and Doyle, 1987). DNA degradation and contamination were monitored on 1% agarose gels. DNA concentration was measured using a Qubit 2.0 fluorometer (Life Technologies, CA, United States). Approximately 1.5 μg of each DNA sample was fragmented by sonication to an average size of 350 bp. Sequencing libraries were generated using the NEBNext Ultra DNA Library Prep Kit for Illumina (NEB, United States) following the manufacturer’s recommendations. Libraries were sequenced using Illumina NovaSeq 6000 (Illumina, San Diego, CA, United States) with 2 × 150 bp paired-end reads.

**TABLE 1:** Taxa, vouchers/references, and GenBank accession numbers used in phylogenetic analyses.

Family	Species	Subgenus	Voucher/references	GenBank accession
Papaveraceae	***C. adunca* Maxim.**	*Cremnocapnos*	Maoxian, Sichuan, Wang et al., 200008	MT920559
	***C. saxicola* Bunting**	*Sophorocapnos*	Zigui, Hubei, Wang et al., 160219	MT920562
	***C. hsiaowutaishanensis* T. P. Wang**	*Corydalis*	Diebu, Gansu, Wang et al., 140388	MT920561
	***C. davidii* Franch.**	*Corydalis*	Baoxing, Sichuan, Wang et al., 140275	MT920560
	*C. inopinata* Prain ex Fedde	*Corydalis*	Langkazi, Xizang, Wang et al., 140599	MT755641
	*C. conspersa* Maxim.	*Corydalis*	Wu et al., 2020	NC_047208
	*L. spectabilis* (L.) Fukuhara	–	Park et al., 2018	NC_039756
	*Chelidonium majus* L.	–	Shi et al., 2019	NC_046829
	*Coreanomecon hylomeconoides* Nakai	–	Kim and Kim, 2016	NC_031446
	*Hylomecon japonica* (Thunb.) Prantl and Kündig	–	Zhang et al., 2019	NC_045388
	*Macleaya microcarpa* (Maxim.) Fedde	–	Zeng et al., 2018	NC_039623
	*Meconopsis racemosa* Maxim.	–	Zeng et al., 2018	NC_039625
	*Papaver somniferum* L.	–	Sun et al., 2016	NC_029434
Circaeasteraceae	*K. uniflora* Balf. f. et W. W. Smith	–	Sun et al., 2017	NC_035873
Lardizabalaceae	*Akebia quinata* (Thunb. ex Houtt.) Decne.	–	Li et al., 2016	NC_033913
Menispermaceae	*Stephania japonica* (Thunb.) Miers	–	Sun et al., 2016	NC_029432
Ranunculaceae	*Glaucidium palmatum* Siebold and Zucc.	–	Zhai et al., 2019	NC_041539
	*Ranunculus cantoniensis* DC.	–	Li et al., 2019	NC_045920
Berberidaceae	*Mahonia bealei* (Fortune) Carrière	–	Ma et al., 2013	NC_022457
	*Nandina domestica* Thunb.	–	Moore et al., 2006	NC_008336
Eupteleaceae	*E. pleiosperma* Hook. f. et Thoms.	–	Sun et al., 2016	NC_029429

*Species in bold are newly sequenced.*

Together with the above four newly sequenced samples, we included another two previously published *Corydalis* plastomes in our analyses (*Corydalis conspersa* Maxim., NC_047208; Wu et al., 2020; and *Corydalis inopinata* Prain ex Fedde MT755641; Xu and Wang, 2020). Another five already-reported *Corydalis* plastomes were not included, because the one plastome of *Corydalis trisecta* Franch. (MK713939; Kanwal et al., 2020) was assigned as unverified in the National Center for Biotechnology Information (NCBI), and the other four plastomes from *C. saxicola* and *Corydalis tomentella* Franch. sequenced in a preprint paper (Ren et al., 2020) may have not sustained to final verification. Besides, the rest of the Ranunculales plastomes (267, excluding unverified sequences and source sequences of the reference sequences) in NCBI (accessed on June 5, 2020) were also included in our analyses. In total, 273 plastomes were used in the present study.

### Plastome Assembly and Annotation

The adaptors in raw data were removed, and low-quality sequences were trimmed using fastp v0.20.1 (Chen et al., 2018) with default parameters. Read quality of clean reads was assessed using FastQC v0.11.9 (Andrews, 2010). The clean reads were assembled using GetOrganelle v1.6.2 (Jin et al., 2018) with the chloroplast genome of *C. inopinata* (MT755641) as reference. Another assembly for each *Corydalis* species was performed using NOVOPlasty (Dierckxsens et al., 2017), with *matK* sequence (MH319908) of *Corydalis temulifolia* as seed, to confirm the GetOrganelle assemblies. The clean reads were mapped to the draft genome using BWA v0.7.17 (Li, 2013), filtered using SAMtools 1.10 (Li et al., 2009), and visualized using IGV 2.8.0 (Robinson et al., 2011) to check the concatenation of contigs. Furthermore, the rearrangements and quadripartite junction sites in the four newly sequenced *Corydalis* plastomes were verified with PCR and Sanger sequencing. The newly designed primers and their location on the plastomes were listed in Supplementary Table S1.

The complete chloroplast genomes were annotated using PGA (Qu et al., 2019), with the plastome of *Euptelea pleiosperma* Hook. f. et Thoms. (NC_029429), *Papaver somniferum* L. (NC_029434), and *Lamprocapnos spectabilis* (L.) Fukuhara (NC_039756) as reference. All tRNAs were verified by ARAGORN v1.2.38 (Laslett and Canback, 2004) and tRNAscan-SE v2.0 (Lowe and Chan, 2016). The annotation results were contrasted with the three annotation reference plastomes and with another three model plant plastomes [*Arabidopsis thaliana* (L.) Heynh., NC_000932; *Nicotiana tabacum* L., NC_001879; and *Gossypium hirsutum* L., NC_007944] and adjusted when necessary. The schematic diagrams of the chloroplast genomes were drawn in OGDRAW v1.3.1 (Greiner et al., 2019). All the newly annotated genomes were submitted to NCBI, and the accession numbers are shown in Table 1.

The GC content, large single-copy (LSC) size, inverted repeat (IR) size, small single-copy (SSC) size, and total genome size of all the 273 plastomes used in the present study were counted using Biopython v1.77 (Cock et al., 2009) and drawn in boxplot using Matplotlib v3.2.1 (Hunter, 2007). In addition, we also calculated GC content of each of the 97 genes (not including intron) shared between *Corydalis* plastomes and other representative Ranunculales plastomes which are listed in Table 1.

### Genome Structure Analyses

To determine synteny and identify possible rearrangements, we compared the four newly sequenced and two previously published *Corydalis* plastomes (those six plastomes were hereafter referred to as “the *Corydalis* plastomes”) with the plastome of *E. pleiosperma* (NC_029429) using Mauve 2.4.0 (Darling et al., 2004) with the “progressiveMauve” algorithm. We employed *E. pleiosperma* as reference, because it exhibited typical angiosperm quadripartite plastome structure and was sister to the remaining Ranunculales. The Mauve result was manually modified to make the notable rearrangements clear and concise. To assess the expansion/contraction of the IR regions in detail, we compared the single-copy (SC)/IR junctions and their adjacent genes of the *Corydalis* plastomes with the *E. pleiosperma* plastome. The schematic diagram was manually modified on the basis of the plastome gene map drawn in OGDRAW. The whole sequence similarity among the *Corydalis* plastomes was comparatively analyzed and plotted using mVISTA (Frazer et al., 2004) in Shuffle-LAGAN mode, with default parameters and with the plastome of *C. conspersa* (NC_047208) as reference.

### Repetitive Sequence Analyses

Three types of repetitive sequence were searched for in the *Corydalis* plastomes, that is, simple sequence repeats (SSR), tandem repeat, and dispersed repeat. Before those analyses were performed, one copy of the IR (IRa) was removed. The SSRs were detected using MISA v2.0 (Thiel et al., 2003) with the minimum numbers of repeats set to 10, 6, 5, 5, 5, and 5 for mononucleotide, dinucleotide, trinucleotide, tetranucleotide, pentanucleotide, and hexanucleotide repeats, respectively. Tandem repeats were detected using the Tandem Repeats Finder v4.09 (Benson, 1999). The alignment parameters match, mismatch, indel, minimum alignment score, and maximum period size were set to 2, 7, 7, 80, and 500, respectively. Four types of dispersed repeat (forward, reverse, complement, and palindromic) were detected using REPuter (Kurtz et al., 2001) with the minimal repeat size set to 30 bp and with a Hamming distance of 1.

### Positive Selection Analyses

To investigate selection pressures from the environment on the *Corydalis* plastomes, we calculated non-synonymous (Ka), synonymous (Ks), and Ka/Ks ratios of 66 protein coding genes shared among the *Corydalis* plastomes, with the plastome of *L. spectabilis* (NC_039756) as reference. *L. spectabilis* was chosen as the reference, because it is closest to *Corydalis* among the taxa whose complete plastome have been reported. The protein-coding DNA sequences were extracted and translated into protein sequences using Biopython v1.77 (Cock et al., 2009). Protein sequences of each gene were aligned using MAFFT v7.450 (Katoh and Standley, 2013) and then converted into their corresponding codon-based nucleotide alignments by the Perl script PAL2NAL v14 (Suyama et al., 2006). Then Ka, Ks, and Ka/Ks ratios were calculated using the KaKs Calculator version 2.0 (Wang et al., 2010) with default parameters, except that we used the 11th genetic code (-c 11). The two anomalously large Ka/Ks values (*K*a/*K*s = 50), which represent extremely low synonymous substitutions in the alignment, were changed to 0. The ratios Ka/Ks > 1, Ka/Ks = 1, and Ka/Ks < 1 suggest positive selection, neutral selection, and purifying selection, respectively.

### Phylogenetic Analyses

In total, 21 plastomes were included in our phylogenetic analyses, including all six *Corydalis* plastomes and 15 representatives of Ranunculales plastomes (Table 1). Among them, *E. pleiosperma* (NC_029429), which was sister to the rest of the Ranunculales, was used as an outgroup to root the trees. The data matrix contains coding regions of 64 protein-coding genes and four rRNA genes, which were shared among the 21 plastomes. Those genes including *infA* gene, *rps15* gene, and the genes listed in Table 3 were excluded, because of pseudogenization or loss of the genes in one or more analyzed plastomes. Codon-based nucleotide alignments of each individual genes were aligned using MAFFT v7.450 (Katoh and Standley, 2013) and PAL2NAL v14 (Suyama et al., 2006) collectively. After manual adjustment, the nucleotide alignments were concatenated. The optimal partitioning scheme and corresponding best fit substitution model for Bayesian inference (BI) analysis were determined using PartitionFinder v2.1.1 (AICc criterion and greedy algorithm for all the mrbayes models, Lanfear et al., 2016) with the concatenated dataset initially partitioned by gene and codon position. Another three similar PartitionFinder analyses was lunched with only one model, GTR, GTR + G, or GTR + I + G; the resulting partitioning schemes with the lowest AICc values were used in maximum likelihood (ML) analysis. Three different phylogenetic methods, that is, BI, ML, and maximum parsimony (MP), were performed. The BI analysis was performed using MrBayes v3.2.7 (Ronquist et al., 2012), with the partitioned dataset and corresponding substitution model, one cold and three heated chains for one-million-generation Markov chains, sampling every 100 generations, and 25% discarded as burn-in. The ML analysis was performed using RAxML v8.2.12 (Stamatakis, 2014) with the partitioned dataset, GTRGAMMA model, and 1,000 bootstrap replicates. The MP analysis was performed using PAUP 4.0a166 (Swofford, 2003), with the directly concatenated dataset, heuristic search with 1,000 random taxon stepwise addition sequences, tree bisection reconnection branch swapping, and 1,000 bootstrap replications.

## Results

### Sequencing Results and Characteristics of the Newly Sequenced *Corydalis* Plastomes

**FIGURE 1:**
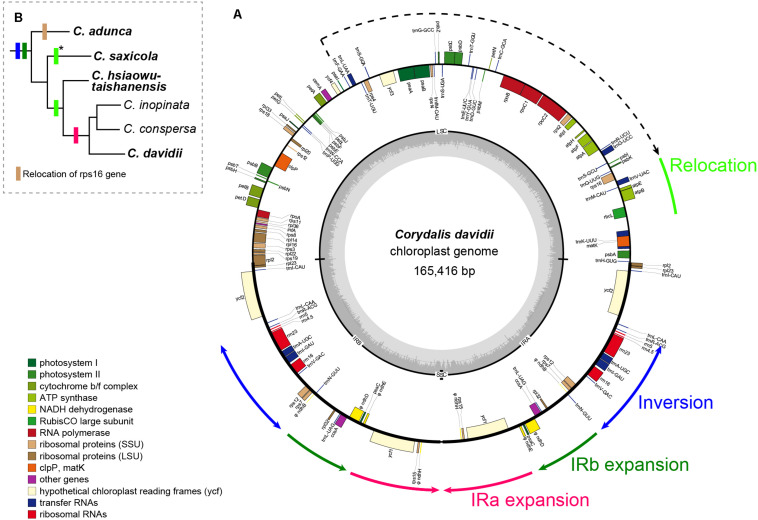
**(A)** Circular gene map of *C. davidii*. Genes inside and outside the map are transcribed in clockwise and counterclockwise directions, respectively. Genes belonging to different functional groups are color coded. Tick lines indicate the extent of the inverted repeats (IRA and IRB). The ring of bar graphs on the inner circle indicates the GC content in dark gray, with the line representing 50%. **(B)** Distributions of rearrangements in other *Corydalis* species. Species in bold are newly sequenced. Color of the boxes is the same with the rearrangements annotated around the circular gene map or indicated under the phylogenetic tree. Asterisks indicate a similar relocation, but downstream of the *atpH* gene.

**FIGURE 2:**
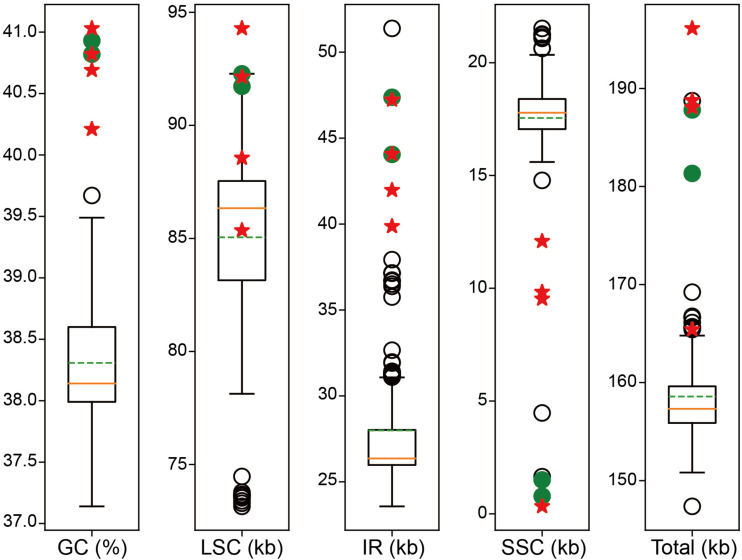
Boxplot distribution of GC content, LSC size, IR size, SSC size, and total genome size of the *Corydalis* plastomes relative to Ranunculales. The orange solid lines indicate the median. The green dotted lines indicate the mean value. The red five-pointed stars indicate the newly sequenced *Corydalis* plastomes. The green solid circles indicate previously published *Corydalis* plastomes. Empty circles indicate the rest outliers.

For the four newly sampled *Corydalis* species, 18,463,522–22,599,330 raw 150 nt paired-end reads (Q30 > 90%) were obtained, with their average coverage depth ranging from 1,273 to 7,154-folds (Table 2). The plastomes assembled using GetOrganelle and NOVOPlasty were identical. All the Sanger sequencing results were also identical to the corresponding sequences of the assembled plastomes. The four newly sequenced complete plastomes ranged in length from 165,416 to 196,128 bp (Table 2). The four newly sequenced *Corydalis* plastomes exhibited the typical angiosperm quadripartite structure, with two IR regions (39,867–47,226 bp each) separating the LSC (85,352–94,289 bp) and SSC (330–12,086 bp) regions. The chloroplast genome schematic diagram of *C. davidii* is taken as an example and shown in Figure 1. The GC content of the four newly sequenced *Corydalis* plastomes ranged from 40.21 to 41.03% (Table 2). As viewed from the boxplots, the GC content, LSC size (except for *C. davidii* and *C. hsiaowutaishanensis*), IR size, and total genome size (except for *C. davidii*) of the four newly sequenced *Corydalis* plastomes were all among the largest, while their SSC sizes were all among the smallest within Ranunculales (Figure 2). The GC content of *C. adunca* (41.03%) is the highest, the LSC size of *C. saxicola* (94,289 bp) is the largest, and the SSC size of *C. davidii* (330 bp) is the smallest ever recorded in this order. For the 97 shared genes in the four newly sequenced *Corydalis* plastomes, 71.65% (278 out of 388) exhibited elevated GC content, as compared with the average GC content of 15 representatives of Ranunculales plastomes (Supplementary Table S2). The four newly sequenced *Corydalis* plastomes contained 101–112 unique genes, consisting of 67–78 protein-coding genes, 30 tRNA genes, and four rRNA genes, and 0–7 pseudogenes (Table 2). In total, 13 genes (*accD*, *clpP*, and 11 *ndh* genes) were detected to be pseudogenized or lost in one or more *Corydalis* plastomes (Table 3). The *accD* gene was lost in the four newly sequenced *Corydalis* plastomes, and the *clpP* gene was lost in *C. hsiaowutaishanensis*. *C. adunca* contained truncated versions of *ndhC*, *ndhB*, *ndhF*, *ndhD*, *ndhI*, *ndhH*, and *ndhA*. *C. davidii* contained truncated versions of *ndhB*, *ndhD*, *ndhE*, and *ndhH* but lacked sequences for *ndhJ*, *ndhK*, *ndhC*, *ndhF*, *ndhG*, *ndhI*, and *ndhA*. Besides, the *rrn16* gene was duplicated in *C. adunca*, and the *psaI* gene was duplicated in both *C. saxicola* and *C. hsiaowutaishanensis*.

**TABLE 2:** Sequencing results (raw data), genome structure, and gene content of the four newly sequenced *Corydalis* plastomes.

Species	Total	Total	Q30	Average	Genome	LSC	IR	SSC	GC	Coding	tRNA	rRNA	Pseudo
	reads	base (G)		depth	size (bp)	(bp)	(bp)	(bp)	content	gene			gene
*C. adunca*	18,463,522	5.54	91.48%	5,316	196,128	92,145	47,226	9,531	41.03%	71	30	4	7
*C. saxicola*	21,189,298	6.36	92.86%	7,154	188,060	94,289	41,969	9,833	40.21%	78	30	4	0
*C. hsiaowutaishanensis*	19,924,311	5.98	90.03%	6,388	188,784	88,558	44,070	12,086	40.82%	77	30	4	0
*C. davidii*	22,599,330	6.78	91.91%	1,273	165,416	85,352	39,867	330	40.69%	67	30	4	4

**TABLE 3:** Pseudogenes and lost genes in the *Corydalis* plastomes.

Species	*ndhJ*	*ndhK*	*ndhC*	*ndhB*	*ndhF*	*ndhD*	*ndhE*	*ndhG*	*ndhI*	*ndhH*	*ndhA*	*accD*	*clpP*	*trnV-UAC*
***C. adunca***	+	+	φ	φ	φ	φ	+	+	φ	φ	φ	−	+	+
***C. saxicola***	+	+	+	+	+	+	+	+	+	+	+	−	+	+
***C. hsiaowutaishanensis***	+	+	+	+	+	+	+	+	+	+	+	−	−	+
***C. davidii***	−	−	−	φ	−	φ	φ	−	−	φ	−	−	+	+
*C. inopinata*	−	φ	φ	φ	φ	φ	+	φ	−	φ	φ	−	+	−
*C. conspersa*	−	−	−	+	+	φ	φ	+	φ	φ	+	−	+	+

*Species in bold are newly sequenced. φ, pseudogene; −, lost gene; +, normal gene.*

### Genome Structure Variations

**FIGURE 3:**
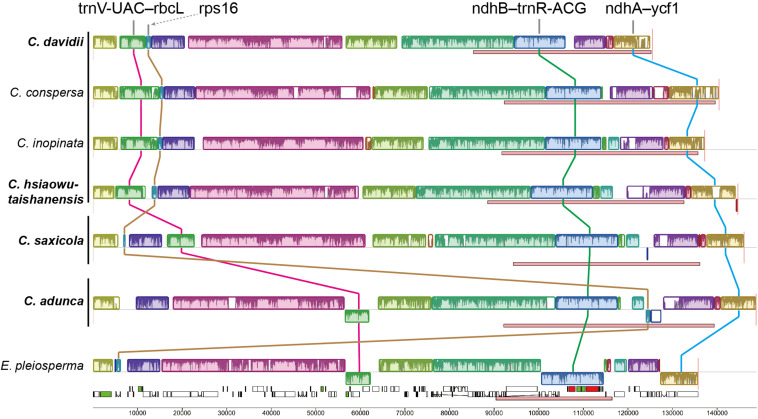
Structural alignments of *Corydalis* and *E. pleiosperma* plastomes using Mauve. Colored blocks represent locally collinear blocks (LCBs), and the blocks connected by lines indicate homology. Blocks drawn below the horizontal line indicate sequences found in an inverted orientation. Pink boxes below each plastome indicate IR regions. The vertical lines after species names, from top to bottom, indicate subg. *Corydalis*, subg. *Sophorocapnos*, and subg. *Cremnocapnos*, respectively. Species in bold are newly sequenced.

Mauve alignment identified 16 locally collinear blocks, from which five unusual rearrangements in the four newly sequenced *Corydalis* plastomes were deduced (Figure 3).

Firstly, one block (∼6–7 kb) contains five genes (*trnV-UAC*, *trnM-CAU*, *atpE*, *atpB*, and *rbcL*) and the associated non-coding sequences, relocated from the typically posterior part of the LSC region to the front. In *C. adunca* (subg. *Cremnocapnos*), those five genes displayed a typical angiosperm location, within the 56–62 kb region, downstream of the *ndhC* gene. In *C. saxicola* (subg. *Sophorocapnos*), those five genes relocated to the 17–23 kb region, downstream of the *atpH* gene. In *C. davidii* and *C. hsiaowutaishanensis* (subg. *Corydalis*), those five genes relocated to the 5–12 kb region, downstream of the *trnK-UUU* gene.

Secondly, one small block (∼1 kb) contained only the *rps16* gene relocated from the LSC region to downstream of the *ndhF* gene in the IR region in *C. adunca*. Meanwhile, one duplicate of the *rrn16* gene in *C. adunca* transferred to the original *rps16* gene loci (not shown in the Mauve result).

Thirdly, one block (∼11–14 kb) in the IR region contains 11 genes (*ndhB*-*trnR-ACG*), inverted uniformly in the four newly sequenced *Corydalis* plastomes.

**FIGURE 4:**
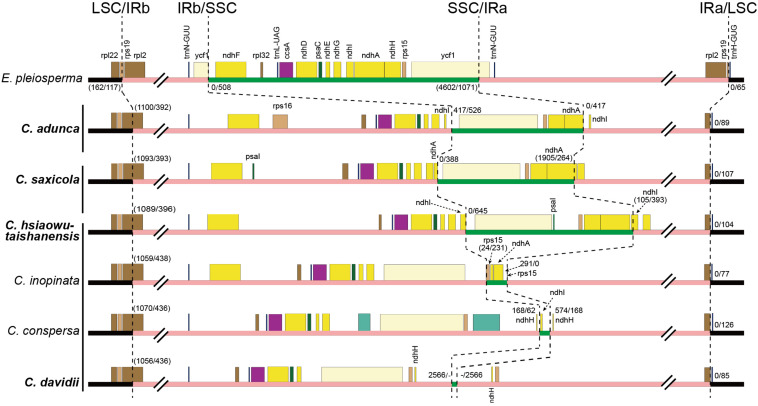
Comparison of LSC, IR, and SSC boundaries among *Corydalis* and *E. pleiosperma* plastomes. The black, pink, and green thick lines indicate LSC, IR, and SSC, respectively. The numbers before and after the slash indicate the distance of the nearest genes relative to the junction site or the length of the front and latter parts of the genes that span the junction site when they appeared within a parenthesis. The vertical lines after species names, from top to bottom, indicate subg. *Cremnocapnos*, subg. *Sophorocapnos*, and subg. *Corydalis*, respectively. Species in bold are newly sequenced.

Fourthly, the IRs expanded greatly, with the absorption of more than half of the SSC region in different types (Figure 4). The IRb region expanded downstream of the pseudogenized *ndhI* gene in *C. adunca* or the middle of the *ndhA* gene in *C. saxicola* or the middle of the *ndhI* gene in *C. hsiaowutaishanensis*, resulting in the transfer of 9–10 genes (though some were pseudogenized) from the SSC region to the IR region. In contrast, in *C. davidii*, IRb expanded downstream of the pseudogenized *ndhE* gene, and successively, IRa expanded downstream of the pseudogenized *ndhH* gene. As a result, the IR regions of the four newly sequenced *Corydalis* plastomes ranged from 39,867 to 44,070 bp in length, which were more than 10 kb longer than the average (28,127 bp) of Ranunculales plastomes.

Lastly, the SSC regions were inverted uniformly in the four newly sequenced *Corydalis* plastomes.

Except for the IR–SSC boundary variations mentioned above, the IR shrank slightly at the LSC/IRb border, from the middle of the *rps19* gene to the middle of the *rpl2* gene (Figure 4), creating a 392–436 bp truncated *rpl2* gene copy (pseudogene) in the IRa region of the four newly sequenced *Corydalis* plastomes.

**FIGURE 5:**
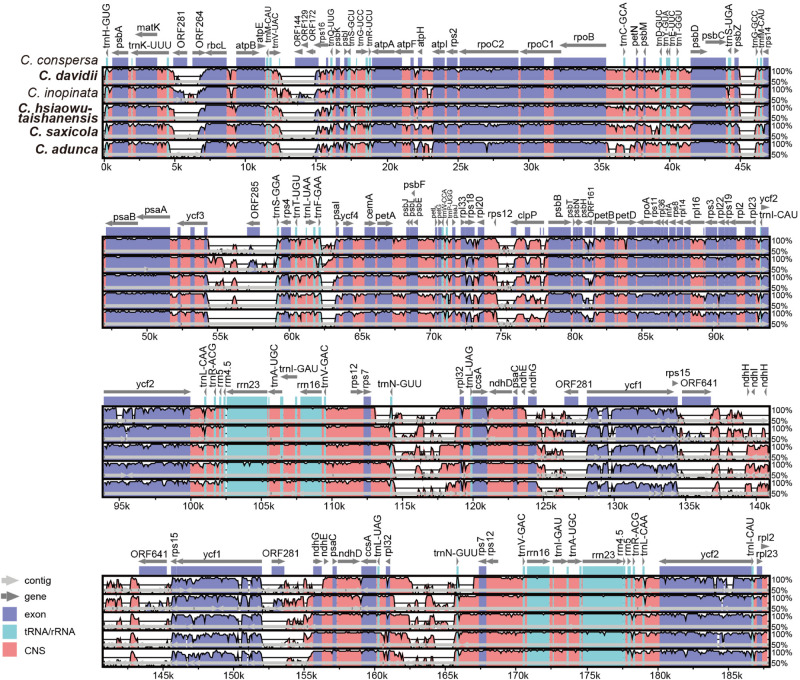
Comparative analyses of plastome differences in the four *Corydalis* plastomes. The dark blue regions represent exons, light-blue regions represent untranslated regions (tRNA and rRNA), and pink regions represent non-coding sequences (CNS). The vertical scale shows the percentage of identity, varying from 50 to 100%. Species in bold are newly sequenced.

The alignment and conserved regions of the *Corydalis* plastomes are shown in Figure 5. Overall, they exhibited high similarity, especially in the gene regions, with the exception that some specific regions that involved plastome rearrangements were highly divergent. For the detected conserved regions, the average identity values of untranslated (tRNA and rRNA), exon, intron, and intergenic regions were 99.22, 93.33, 93.0, and 88.27%, respectively. Some intron regions (*rps16*, *rpl16*, and *trnK-UUU*) and intergenic regions (*trnQ-UUG*-*psbK*, *psbK*-*psbI*, *atpH*-*atpI*, *rpoB*-*trnC-GCA*, *trnC-GCA*-*petN*, *psbM*-*trnD-GUC*, *trnD-GUC*-*trnY-GUA*, *trnE-UUC*-*trnT-GGU*, *trnT-GGU*-*psbD*, *trnT-UGU*-*trnL-UAA*, *rbcL*-*atpB*, *psaI*-*ycf4*, *petA*-*psbJ*, and *psbE*-*petL*), which exhibited some extent of divergence, were identified, and they have the potential to be DNA markers in future phylogenetic analyses.

### Repeat Sequences

In total, we detected 150 SSRs including 135 mononucleotide repeats and 15 dinucleotide repeats in the four newly sequenced *Corydalis* plastomes (not including IRa; Table 4). Almost all mononucleotide repeats were composed of A/T (94.07%), while only 5.93% were composed of C/G. The 15 dinucleotide repeats consisted of 14 AT/TA repeats and one AG repeat. The total numbers of SSRs in the four newly sequenced *Corydalis* plastomes were similar to those of *C. inopinata*, *C. conspersa*, and *L. spectabilis*. It was, however, larger than that of *P. somniferum* and smaller than that of *E. pleiosperma*. In total, 26–28 tandem repeats and 156–274 dispersed repeats were detected in each of the four newly sequenced *Corydalis* plastomes (Table 4). *C. adunca* has the most dispersed repeats (274), which were ∼16 times of *E. pleiosperma* (17) and ∼30 times of *P. somniferum* (9).

**TABLE 4:** Numbers of SSR, tandem repeat, and dispersed repeat in the plastomes of *Corydalis* species and related species.

Species	Simple sequence repeat											Tandem repeat	Dispersed repeat				Total
	A	C	G	T	AT	TA	AG	TG	AAT	GAG	TTC		Forward	Reverse	Complement	Palindr	
										GAT	GGA					omic	
***C. adunca***	20	1	–	15	2	1	1	–	–	–	–	27	137	9	4	124	341
***C. saxicola***	7	2	–	21	2	3	–	–	–	–	–	26	180	–	–	15	256
***C. hsiaowutaishanensis***	13	3	1	16	2	1	–	–	–	–	–	28	121	–	–	35	220
***C. davidii***	24	1	–	11	1	2	–	–	–	–	–	28	188	1	–	2	258
*C. inopinata*	15	2	–	9	1	–	–	–	–	–	–	19	76	–	–	5	127
*C. conspersa*	11	1	–	13	1	–	–	–	–	–	–	29	276	–	–	4	335
*L. spectabilis*	17	1	1	23	1	–	–	1	–	1	1	19	83	–	–	4	152
*P. somniferum*	7	–	–	8	–	–	–	–	–	–	–	5	6	–	–	3	29
*E. pleiosperma*	33	–	2	35	1	2	–	–	1	–	–	31	11	1	–	5	122

*Species in bold are newly sequenced.*

### Nucleotide Substitution Rates

**FIGURE 6:**
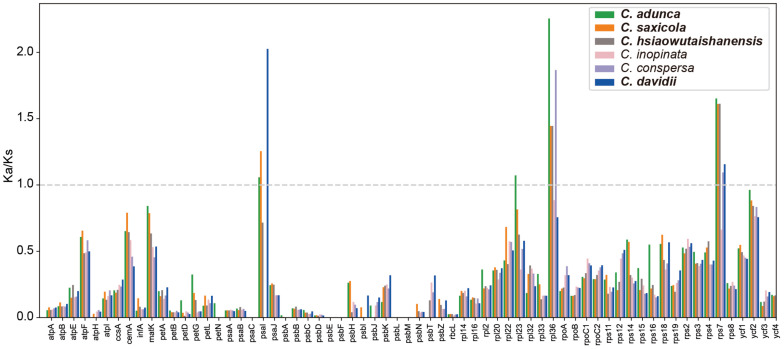
The Ka/Ks ratios of 66 protein-coding genes of the *Corydalis* plastomes relative to *L. spectabilis*. Species in bold are newly sequenced.

The Ka/Ks ratios of the 66 protein-coding genes are shown in the bar graph (Figure 6). The majority (85.1%) of Ka/Ks ratios was between 0 and 0.5. The average Ka/Ks ratio for all those genes was 0.26. Higher Ka/Ks values were detected mainly in the *rpl* and *rps* gene families. The Ka/Ks ratios of four genes (*psaI*, *rpl23*, *rpl36*, and *rps7*) were greater than 1 in one or more pairwise comparisons. In total, 13 Ka/Ks ratios, that is, those of all the four genes in *C. adunca*; *psaI*, *rpl36*, and *rps7* in *C. saxicola*; *rpl36* and *rps7* in both *C. hsiaowutaishanensis* and *C. conspersa*; and *psaI* and *rps7* in *C. davidii*, were larger than 1.

### Phylogenetic Analyses

**FIGURE 7:**
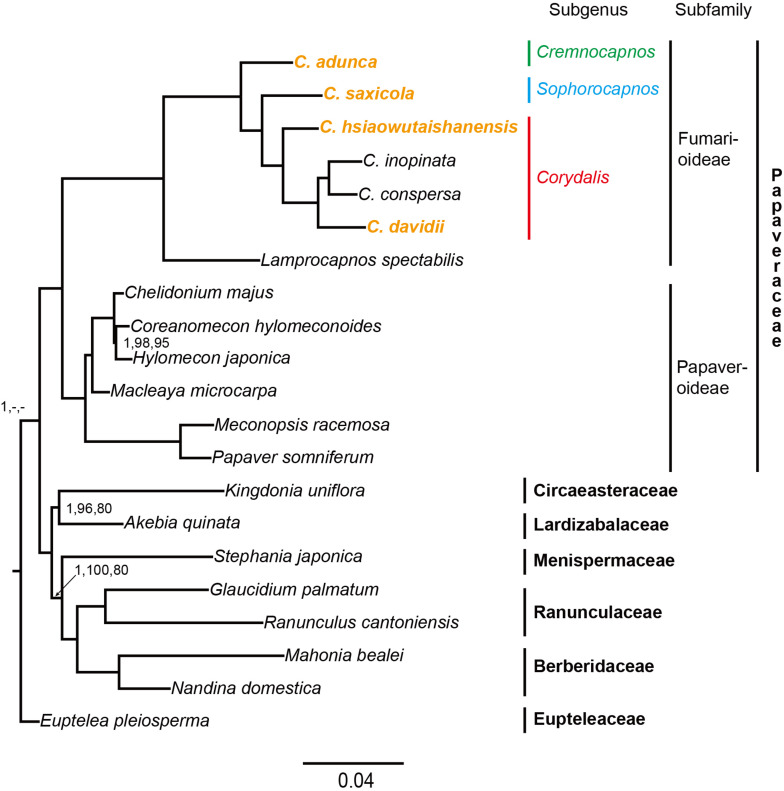
Phylogenetic tree conducted using BI methods. The numbers above branches represent BI posterior probability, ML bootstrap support, and MP bootstrap support, respectively. The full support values were not indicated.

The data matrix used in phylogenetic analyses consisted of 63,027 nucleotide sites; of these, 8,642 (13.7%) were parsimony informative. The PartitionFinder2 analyses using mrbayes models partitioned the concatenated dataset into 70 subsets and 14 substitution models (Supplementary Table S3). The AICc values of another three independent PartitionFinder2 analyses using GTR, GTR + G, or GTR + I + G were 524,755.977691, 518,714.822997, and 518,513.525426, respectively. The partitioning scheme determined using GTR + I + G (62 subsets; Supplementary Table S4) has the lowest AICc value and was used in ML analysis. The three phylogenetic analyses (BI, ML, and MP) generated identical topologies, and the vast majority branches received full support. Thus, only the BI phylogenetic tree is presented in Figure 7. Three clades can be defined in the phylogenetic tree, that is, the Eupteleaceae clade, the Papaveraceae clade, and the clade composed of the rest of the Ranunculales families. Within Papaveraceae, two sub-clades corresponding to the subfamilies (Papaveroideae and Fumarioideae) were resolved with full support. *Corydalis* belongs to the Fumarioideae sub-clade, and all lineages within *Corydalis* were fully supported. The four newly sequenced *Corydalis* species, that is, *C. adunca* (sect. *Strictae*), *C. saxicola* (sect. *Thalictrifoliae*), *C. hsiaowutaishanensis* (sect. *Dactylotuber*), and *C. davidii* (sect. *Davidianae*), served as the successively diverged lineages in this genus.

## Discussion

### Abnormal Genome Sizes, Reduced Gene Composition, Elevated GC Content, and High Number of Repeat Sequences

The LSC size, IR size, SSC size, and total genome size of the *Corydalis* plastomes were proven to be abnormally larger or smaller than those of the other Ranunculales, though a few exceptions existed (Figure 2). Several factors, for example, gene duplications, gene losses, and IR expansions, may be responsible for the variation in *Corydalis* plastome sizes. Among them, the expansions of IR into the SSC region (for details, see section “Various IR Expansions”) have contributed the most to the increase of plastome size. Gene losses (and/or pseudogenizations), on the contrary, have partially counteracted the increase of plastome size. Owing to its pseudogenization or loss of all eight *ndh* genes in the SSC and IR regions, *C. davidii* had the smallest IR and total plastome size, albeit its IR expansion was most pronounced in *Corydalis*.

With respect to gene content, the *Corydalis* plastomes exhibited extensive pseudogenization or loss of some of the 13 genes (11 *ndh*, *accD*, and *clpP* genes). The 11 chloroplast *ndh* genes that encode NADH dehydrogenase subunits are essential for the function of chloroplast and are widely present in angiosperms (Martin and Sabater, 2010). However, we found that 7–11 of the *ndh* genes were either pseudogenized or lost in the plastome of *C. adunca*, *C. davidii*, *C. inopinata*, and *C. conspersa*. The pseudogenization and/or loss of *ndhJ*, *ndhK*, and *ndhC* were possibly associated with the adjacent relocation event in the LSC region, while the pseudogenization and/or loss of the rest of the *ndh* genes were possibly associated with the IR boundary shift, as evidenced in another Ranunculales species (*Kingdonia uniflora*, Sun et al., 2017), where similar plastome rearrangements were also accompanied by pseudogenization and/or loss of *ndh* genes. In a phylogenetic perspective, the pseudogenization or loss of *ndh* genes in the *Corydalis* and Ranunculales may have occurred independently after the split of those species, which was probably a similar case found in the research of Orchidaceae (Lin et al., 2015). It is necessary to use more species to elucidate the pattern and mechanism of pseudogenization and loss of *ndh* genes within *Corydalis*. Another unusual case is the uniform loss of the *accD* gene in the *Corydalis* plastomes. We speculate that the loss of the *accD* gene probably occurred in the common ancestor of the *Corydalis* species. The *accD* gene encodes one of four subunits of the acetyl-CoA carboxylase enzyme (ACC), which is a rate-limiting enzyme in the first committed step of fatty acid synthesis (Cronan and Waldrop, 2002; Sasaki and Nagano, 2004). The loss of the *accD* gene is lethal, as evidenced by the study of tobacco (Kode et al., 2005). Within angiosperms, the lost *accD* has been found to relocate to the nucleus in some species, such as *Trifolium repens* (Magee et al., 2010), some Campanulaceae species (Rousseau et al., 2013), and *Platycodon grandiflorum* (Hong et al., 2017). We are not sure whether the plastid-encoded *accD* gene is lost entirely or functionally transferred to the nucleus in *Corydalis*, but there must be some kind of compensatory mechanism that can explain their loss from the plastome.

The GC content of the *Corydalis* plastomes (40.21–41.03%) was slightly higher than the average of Ranunculales (38.31%). It seems not unusual, but when examining the NCBI RefSeq database (accessed on October 19, 2020), we can find that only ∼130 plastomes out of more than 5,000 land plant plastomes have GC content higher than 40%. The increase of GC content in the *Corydalis* plastomes has affected not only the gene regions but also the intergenic and intron regions. This is because the GC content of the gene region and intergenic and intron regions of the *Corydalis* plastomes has increased, by 0.8–1.75% and 3.32–4.76%, respectively, as compared with the average of the 15 representatives of Ranunculales plastomes. It is well known that the GC base pair is more stable than the AT. Thus, the overall increase of GC content may potentially enhance the stability of the plastomes and consequently contribute to their adaptation to some harsh conditions. Here, the two largest GC contents within Ranunculales, kept by *C. adunca* and *C. inopinata*, may have contributed to their adaptation to the Qinghai–Tibet Plateau.

The pairwise Ka/Ks ratios were widely used as an effective way to detect positive selection or adaptive evolution in plant species (Wang et al., 2010; Gao et al., 2019). For the protein-coding genes in the *Corydalis* plastomes, the majority (85.1%) of Ka/Ks ratios obtained in our analyses were between 0 and 0.5, which was consistent with the result of previous researches (Nei et al., 2010; Yang et al., 2020). This suggests that the majority of genes in the *Corydalis* plastomes were probably under purifying selection. The *rpl* and *rps* gene families, which are involved in self-replication, showed higher Ka/Ks ratios compared with the majority of the rest of the genes in the *Corydalis* plastome. This may contribute to their adaptation to diverse habitats. In the present study, only four genes (*psaI*, *rpl23*, *rpl36*, and *rps7*) showed Ka/Ks ratios greater than 1 (Figure 6) in one or more pairwise comparisons, indicating that they may undergo some selective pressure. All four genes (*psaI*, *rpl23*, *rpl36*, and *rps7*) and three genes (except for *rpl23*) in the plastomes of *C. adunca* and *C. saxicola*, respectively, showed Ka/Ks ratios greater than 1. Considering that these two species readily grow in dry habitats, which is different from the rest of the *Corydalis* species and *L. spectabilis*, we speculate that the chloroplast functional genes may have facilitated their adaptation to such a dry environment.

Another unique feature of the *Corydalis* plastomes was its high number of repeat sequences, especially the dispersed repeats (81–280), which can be ∼30 times of the number of the dispersed repeats in *P. somniferum* (9). Some researchers have suggested that repeat sequences may contribute to plastome rearrangement (Ogihara et al., 1988; Milligan et al., 1989; Cole et al., 2018). In the present study, we also detected that the dispersed repeat sequences often located around the loci that involved rearrangement.

### Rare Gene Relocations and Inversions

Among the various chloroplast genome rearrangements, relocation is probably the least common in angiosperms (Mower and Vickrey, 2018). Until now, it was only reported within a few lineages, such as Oleaceae (Lee et al., 2007), Campanulaceae (Knox, 2014; Knox and Li, 2017; Uribe-Convers et al., 2017), and Ranunculaceae (Liu et al., 2018). In the present study, a 5 kb segment containing five genes (*trnV-UAC*-*rbcL*), which were traditionally located downstream of the *ndhC* gene, has unconventionally inverted and relocated either downstream of the *atpH* gene in *C. saxicola* (subg. *Sophorocapnos*) or downstream of the *trnK-UUU* gene in both *C. davidii* and *C. hsiaowutaishanensis* (subg. *Corydalis*) (Figure 3). The latter relocation event was also detected in the two previously published *Corydalis* plastomes (subg. *Corydalis*; Wu et al., 2020; Xu and Wang, 2020). In a phylogenetic view, it seems that the locations of those five genes (*trnV-UAC*-*rbcL*) were subgenus specific, and the relocation event probably occurred after the divergence of subg. *Cremnocapnos*. Moreover, subg. *Sophorocapnos* and subg. *Corydalis* displayed different types of *trnV-UAC-rbcL* location, suggesting that the two relocation events occurred independently in these two subgenera. Until now, the relocation of those five genes (*trnV-UAC*-*rbcL*) was only reported from *Corydalis* within Ranunculales. Research from some Oleaceae species (Lee et al., 2007) revealed that the plastome relocation resulted from two overlapping inversions: the first inversion inverted the entire segment, and the second restored some part to its original gene order. It is possible that the relocation of those five genes (*trnV-UAC*-*rbcL*) in *Corydalis* is also the result of two overlapping inversions. Putatively, the first inversion for subg. *Corydalis* is ∼50 kb, comprising *rps16*-*rbcL*, which translocates *trnV-UAC-rbcL* to downstream of *trnK-UUU*, and the second inversion restores the subsequent genes (*ndhC*-*rps16*) to their original order. Additionally, an inversion of *trnQ-UUG*-*rbcL*, which was similar to the first “putative” subg. *Corydalis* inversion, has been reported in two Ranunculales species (*Circaeaster agrestis* Maxim. and *K. uniflora*, Sun et al., 2017). It is likely that this “putative” inversion may also be found in some *Corydalis* species. There were reports of similar relocations of *trnV-UAC*-*rbcL* to downstream the *psbA* gene in at least 22 genera of Campanulaceae (Knox, 2014; Knox and Li, 2017; Uribe-Convers et al., 2017). Significantly, similar plastome rearrangements have happened independently in those two distantly related lineages. Elucidating the origin, evolution, and mechanism under such similar but independent genome rearrangements seems an exciting topic to explore in further comparative genomic studies.

One segment that contains 11 genes (*ndhB*-*trnR-ACG*) in the IR region, accounting for about 60% of the typical IR region, inverted uniformly in the *Corydalis* plastomes and *L. spectabilis* plastome (Park et al., 2018). This inversion was shared by all the sequenced Fumarioideae plastomes, but it was absent from the subfamily Papaveroideae. This indicated that the inversion has occurred after the divergence of those two Papaveraceae subfamilies and existed in the common ancestor of *Lamprocapnos* and *Corydalis*. In the nearby region of the above inversion, *C. adunca*, which was sister to the rest of the *Corydalis*, was characterized by a unique relocation of the *rps16* gene from the LSC region to the IR region and by the transfer of a replicate of its *rrn16* gene to the typical *rps16* gene site. Whether those two rearrangements occurred in the plastome of *C. adunca* simultaneously or independently, extra hidden plastome rearrangements must have happened in the evolutionary history. *L. spectabilis*, a species that diverged before *Corydalis* within the subfamily Fumarioideae, also exhibited some extra rearrangements around the inverted *ndhB*-*trnR-ACG* segment (Park et al., 2018). Those portend that the common ancestor of *Corydalis* and *Lamprocapnos* may possess a variable IR region. With a variable IR region in the ancestor, it may be interesting to research why only the inversion of the *ndhB-trnR-ACG* segment in the IR regions was kept throughout all those species of the subfamily Fumarioideae.

The inversion in the SSC regions, by the way, has been detected in the *Corydalis* plastomes, as compared with the plastome of *E. pleiosperma* and most other angiosperms. However, it is probably not a noticeable feature, because previous studies have found that most plastomes often occur in two distinct haplotypes (with equal frequency) differing in the orientation of SC regions (Palmer, 1983; Wang and Lanfear, 2019).

### Various IR Expansions

The IR regions of angiosperm plastomes usually start around the *rps19* gene and terminate almost uniformly at either downstream of the *trnN-GUU* or truncated *ycf1* gene. IR expansion has been reported in some lineages, but it was usually into the LSC region (e.g., in Ma et al., 2013; Sun et al., 2013; Liu et al., 2018; Mower and Vickrey, 2018; Zhai et al., 2019). The IR/SSC junctions were thought to be relatively stable (Sun et al., 2016; Zhu et al., 2016; Mower and Vickrey, 2018; Park et al., 2018). In the present study, we found that the IR of the *Corydalis* plastomes expanded markedly at the IR/SSC boundaries (Figures 3, 4), which has resulted in the second to seventh largest IR sizes and the three smallest and sixth to eighth smallest SSC sizes in Ranunculales. The three earlier diverged *Corydalis* species (*C. adunca*, *C. saxicola*, and *C. hsiaowutaishanensis*; Figure 7) have only IRb/SSC expansion, while *C. inopinata*, *C. conspersa*, and *C. davidii* have a further IRa/SSC expansion. *L. spectabilis*, which also belongs to the subfamily Fumarioideae (Papaveraceae) but diverged earlier (Figure 7) than *Corydalis*, was characterized with the expansion of IRa into the SSC region. Thus, three types of IR/SSC expansion can be defined within the subfamily Fumarioideae, that is, IRa/SSC expansion, IRb/SSC expansion, and IRb/SSC + IRa/SSC expansion. While in the subfamily Papaveroideae (Papaveraceae), no IR expansion was observed. Hereby, we can deduce that IR expansion occurred after the diversification of those two Papaveraceae subfamilies. After a further examination of the IR/SSC boundary within *Corydalis*, we found that the adjacent genes differed between lineages. These results suggest that more than one IR expansion event has occurred and that each lineage has its specific IR expansion history. More extensive sampling is needed to elucidate the IR expansion pattern within *Corydalis*.

### Phylogenetic Implications and New DNA Markers

The plastid phylogenomic analyses generated highly supported phylogeny with three distinct clades (i.e., Eupteleaceae, Papaveraceae, and the rest of the Ranunculales families), which is consistent with the results of previous studies (Wang et al., 2009; Pérez-Gutiérrez et al., 2015; Sauquet et al., 2015; Sun et al., 2016). All the *Corydalis* lineages were fully supported in the phylogenetic tree, which indicates that the application of complete plastome data can improve the resolution of the phylogeny of *Corydalis*. This suggests that the complete plastome data have the potential to be employed in the construction of a robust phylogeny for *Corydalis*, which would be instructive for resolving the taxonomic controversy in this genus. Importantly, we resolved *C. adunca* (sect. *Strictae*) to be a lineage with early divergence in this genus and *C. hsiaowutaishanensis* (sect. *Dactylotuber*) to be an earlier diverged lineage in subg. *Corydalis*. This was consistent with the results of previous studies based on two DNA markers (*rps16* and *matK*; Wang, 2006) but contradicted with the morphological character-based taxonomy to some extent (Wu et al., 1996, 1999).

Highly variable DNA markers are useful, especially in fast species identification and wide-range phylogenetic analyses. In the present study, the introns of *rps16*, *rpl16*, and *trnK-UUU* and intergenic regions of *trnQ-UUG*-*psbK*, *psbK*-*psbI*, *atpH*-*atpI*, *rpoB*-*trnC-GCA*, etc., which exhibited some extent of divergence, were identified and have great potential to be exploited as DNA markers. Some of them have already been used as markers in previous phylogenetic analyses of *Corydalis* (e.g., Wang, 2006; Zhang Z.X. et al., 2016; Jiang et al., 2018; Ren et al., 2018), while the rest is in need of exploitation in the future. Nevertheless, they should be used with caution in *Corydalis*, because genes that stayed in the SC region or relocated to the IR region usually obtain different substitution rates (Zhu et al., 2016), which may mislead the phylogenetic reference. For example, the *rps*16 gene, which has been used in the previous phylogenetic analysis of *Corydalis* (Lidén et al., 1997; Wang, 2006), was found to have relocated to IR in *C. adunca*, while it still remained in the LSC region in the rest of the sequenced *Corydalis* species.

## Conclusion

We provided insights into the plastome structure of *Corydalis* and resolved *Corydalis* to be an overlooked lineage that exhibited some extraordinary rearrangements (relocations, inversions, IR expansions, pseudogenizations, gene losses, gene duplications, etc.). Although similar rearrangements have occurred multiple times in the history of angiosperm, it is noteworthy that all those rearrangements existed in a single genus. The plastome data also turned out to be effective in enhancing the resolution of phylogenies in *Corydalis*, and it could be alternatively employed to construct a robust phylogeny for *Corydalis* in further studies. The results obtained in this study may be valuable for further study on the taxonomy, phylogeny, and evolution of *Corydalis*, a taxonomically difficult but fascinating genus.

## Data Availability Statement

The datasets generated for this study can be found in National Center for Biotechnology Information (NCBI) under the accession numbers: MT920559–MT920562.

## Author Contributions

XX performed the analyses and drafted the manuscript. DW provided the suggestions on structuring the manuscript and the main points of the discussion and revised the manuscript. Both authors conceived the study, obtained the samples, designed the experiments, read, and approved the final manuscript.

## Conflict of Interest

The authors declare that the research was conducted in the absence of any commercial or financial relationships that could be construed as a potential conflict of interest.
