# Supplementary material for: Comparative Chloroplast Genomics of Corydalis Species (Papaveraceae): Evolutionary Perspectives on Their Unusual Large Scale Rearrangements
Source: Front Plant Sci. 2021 Jan 27;11:600354. doi: 10.3389/fpls.2020.600354 (PMC7873532; doi:10.3389/fpls.2020.600354)
Supplement: Supplementary file 1 [file Data_Sheet_1.PDF]

Supplementary Table S1. PCR and sequencing primers designed in this study to verify the plastome rearrangements and quadripartite junction sites. The numbers in the columns "Start" and "End" indicate the start and end position of the primer on the plastome. A hyphen "-" indicate that the primer were not used in this species.

Rearranged or quadripartite junction site	Primer name	Primer sequence (5' to 3')	*C. adunca*		*C. saxicola*		*C. hsiaowutai-shanensis*		*C. davidii*		Note
			Start	End	Start	End	Start	End	Start	End	
IRa/LSC junction	*rpl2-psbA*-F	CCCAGAGGGRCKAGAATTGG	196049	196068	187980	187999	188704	188723	165293	165312	-
	rpl2-psbA-R	CTTTCTAGCTGCTTGGCCTG	825	806	794	775	814	795	838	819	-
LSC/IRa junction	*rpl2*-F	CCAYTCCTCTGACTACAGGA	91207	91226	93358	93377	87631	87650	84458	84477	-
	rpl2-R	ATTSTGGGAAAGGGMGGAAT	92431	92412	94576	94557	88845	88826	85682	85663	-
IRb-SSC junction	*IRb-SSC*-F	GGTTTGACTCGCCCGAAGTA	139273	139292	-	-	-	-	-	-	-
	IRb-SSC-R	GGAGGACGCAAACTAAACCG	139457	139438	-	-	-	-	-	-	-
	ndhI-ycf1-F	TGGCCGATATACCTTGCAGC	-	-	135862	135881	-	-	-	-	-
	ndhI-ycf1-R	CCCTGCGGTCAGAAATCCAT	-	-	136748	136729	-	-	-	-	-
	ndhI-ycf1-1F	CTGACATTGGTAACCGACCCA	-	-	-	-	132289	132309	-	-	-
	ndhI-ycf1-1R	CGGCCACAACGACTGAATTG	-	-	-	-	133346	133327	-	-	-
	IRb-SSC-1F	CGCGTTCCCAAGTTCCTAGT	-	-	-	-	-	-	124866	124885	-
	IRb-SSC-1R	CCATTCCCCAGCCGTTAGTT	-	-	-	-	-	-	125501	125482	-
SSC-IRa junction	*ndhA-ndhI*-F	AGAGCTTCCCTTCCCTGTCT	148668	148687	-	-	-	-	-	-	-
	ndhA-ndhI-R	GGTTTGACTCGCCCGAAGTA	149001	148982	-	-	-	-	-	-	-
	ndhA-ndhI-1F	TACGAGCTGCTGCTCAATCG	-	-	144671	144690	-	-	-	-	-
	ndhA-ndhI-1R	CTGACATTGGTAACCGACCCA	-	-	146862	146842	-	-	-	-	same as *ndhI-ycf1*-1F
	ndhA-ndhI-2F	GGGTGAGATCAATTCAGAAGCAC	-	-	-	-	143743	143765	-	-	-

	ndhA-ndhI-2R	CTGACATTGGTAACCGACCCA	-	-	-	-	145054	145034	-	-	-
Inversion in IR region (involve *ndhB–trnR-ACG* genes; upstream side)	*trnL-trnR*-F	CTCCACGCTSTTTAGCACGA	101457	101476	103392	103411	97812	97831	94051	94070	-
	trnL-trnR-R	GAGAGCACGTGGCTACGAA	104075	104057	104120	104102	98545	98527	94776	94758	-
	trnL-trnR-1F	TGAACCACCCATCCATTTTCT	102365	102385	-	-	-	-	-	-	-
	trnL-trnR-1R	TTCTTCATCGGTTCCCAGGC	103065	103046	-	-	-	-	-	-	-
Inversion in IR region (involve *ndhB–trnR-ACG* genes; downstream side)	*ndhB-trnN*-F	TTCCTCCACTCGCAGGTTTT	117247	117266	117379	117398	111710	111729	-	-	-
	ndhB-trnN-R	TCAGTGGTAGAGCGGTCGG	118115	118097	118244	118226	112556	112538	-	-	-
	rps7-trnN-F	AGGCCATACGCAAAAGGGAA	-	-	-	-	-	-	105642	105661	-
	rps7-trnN-R	TCAGTGGTAGAGCGGTCGG	-	-	-	-	-	-	106192	106174	same as *ndhB-trnN*-R
Relocation of *rrn16* gene (downstream side)	*rrn16-psbK*-F	GCCGATGCTTATTCCCCAGA	9031	9050	-	-	-	-	-	-	-
	rrn16-psbK-R	AGCTTGCCAAACAAGGGCTA	10335	10316	-	-	-	-	-	-	-
Relocation of *trnV-UAC–rbcL* genes to after *atpH* gene (upstream side)	*atpH-rbcL*-F	GTACCTTGACCAACTCCGGG	14930	14949	-	-	-	-	-	-	-
	atpH-rbcL-1F	CCGGAGGATTTGGACCTCTC	15850	15869	-	-	-	-	-	-	-
	atpH-rbcL-2F	CCTCGATTGTCGTTTCCCCT	16665	16684	-	-	-	-	-	-	-
	atpH-rbcL-R	CGAGTAGCCCTAGAAGCGTG	17627	17608	-	-	-	-	-	-	pair with the above three Forword primers
Relocation of *trnV-UAC–rbcL* genes to after *atpH* gene (downstream side)	*trnV-atpI*-F	CTGTTTTACCGAGAAAGTCTACG	22613	22635	-	-	-	-	-	-	-
	trnV-atpI-1F	TCTGGTTGCGGTTTCGTAGG	23672	23691	-	-	-	-	-	-	-
	trnV-atpI-R	TCAACCAACGCCAATCCTCT	24882	24863	-	-	-	-	-	-	pair with the above two Forword primers

Relocation of *trnV-UAC–rbcL* genes to after *trnK-UUU* gene (upstream side)	*trnK-rbcL*-F	TAGGCGCACTTAAAAGCCGA	-	-	-	-	4458	4477	-	-	-
	trnK-rbcL-R	CGAGTAGCCCTAGAAGCGTG	-	-	-	-	5852	5833	-	-	-
	trnK-rbcL-1F	TAGGCGCACTTAAAAGCCGA	-	-	-	-	-	-	4481	4500	same as *trnK-rbcL*-F
	trnK-rbcL-1R	GGCTCCCGAGTAAAGGATGG	-	-	-	-	-	-	5559	5540	-
	trnK-rbcL-2F	ATTGGTGGCCGCGATTACAA	-	-	-	-	-	-	5462	5481	-
	trnK-rbcL-2R	GGACGTGATCTTGCTCGTGA	-	-	-	-	-	-	6546	6527	-
Relocation of *trnV-UAC*–rbcL genes to after *trnK-UUU* gene (downstream side)	*trnV-rps16*-F	GGTTCGAATCCGTATAGCCC	-	-	-	-	-	-	11563	11582	-
	trnV-rps16-R	TGATGTTCGATCCCGACGAG	-	-	-	-	-	-	12029	12014	-
	trnV-rps16-1F	GGTTCGAATCCGTATAGCCC	-	-	-	-	10853	10872	-	-	same as *trnV-rps16*-F
	trnV-rps16-1R	CCTTGACCCACTCGACTCTC	-	-	-	-	11917	11898	-	-	-
	trnV-rps16-2F	TGGTTCGACGGTGCCATTAC	-	-	-	-	11788	11807	-	-	-
	trnV-rps16-3F	TTGGGACGAAGAAACAGCGA	-	-	-	-	12613	12632	-	-	-
	trnV-rps16-3R	TGATGTTCGATCCCGACGAG	-	-	-	-	13415	13396	-	-	pair with the above two Forword primers, same as *trnV-rps16*-R
Relocation of *rps16* gene (upstream side)	*ndhF-rps16*-F	CTAAAGGAACCGGAAGCGGA	-	-	-	-	-	-	123130	123149	-
	ndhF-rps16-R	CAAGTCGCACGTTGCTTTCT	-	-	-	-	-	-	124230	124211	-
IRb and IRa expansion junction	*ndhE-ycf1*-F	CGCGCAAATGTCTCCTTTTCA	-	-	-	-	-	-	114124	114144	-
	ndhE-ycf1-R	GGTCCCTGCGGTCAGAAATC	-	-	-	-	-	-	115890	115871	-

Supplementary Table S2. GC content of the 97 genes shared among *Corydalis* plastomes and 15 representative Ranunculales plastomes.

Gene	*C. adunca*	*C. saxicola*	*C. hsiaowutaishanensis*	*C. davidii*	*C. inopinata*	*C. conspersa*	Average (*Corydalis*)	Average (15 Ranunculales)
atpA	42.26	41.80	42.59	42.13	42.32	41.80	42.15	41.58
atpB	43.82	43.66	44.00	44.20	43.93	44.00	43.93	43.27
atpE	39.30	37.84	38.56	40.05	39.80	39.95	39.25	40.21
atpF	39.46	38.56	38.38	39.82	40.36	39.64	39.37	37.61
atpH	46.34	46.34	47.97	45.53	45.53	45.12	46.14	46.59
atpI	39.65	39.25	39.92	39.65	40.46	40.19	39.85	39.12
ccsA	35.71	35.93	35.81	36.43	36.12	36.34	36.06	33.46
cemA	35.36	34.49	34.20	34.35	34.06	33.77	34.37	33.59
matK	35.99	35.79	36.13	35.66	35.72	35.19	35.75	33.66
petA	41.33	39.98	39.77	40.29	40.47	40.77	40.44	40.55
petB	41.51	40.59	41.05	41.36	41.36	41.20	41.18	41.50
petD	40.08	39.48	40.28	40.67	39.48	40.28	40.05	38.76
petG	35.96	36.84	37.72	38.60	37.72	38.60	37.57	36.20
petL	39.58	36.46	38.54	39.58	39.58	40.63	39.06	36.88
petN	38.89	41.11	40.00	40.00	40.00	40.00	40.00	40.67
psaA	44.30	44.39	44.12	44.18	43.96	44.25	44.20	43.52
psaB	42.31	41.77	42.13	42.90	42.95	42.90	42.49	41.77
psaC	43.90	43.50	43.50	43.90	43.50	42.68	43.50	43.01
psaI	36.04	38.74	39.64	38.74	40.54	41.44	39.19	35.80
psaJ	43.26	42.55	43.26	43.97	43.97	43.97	43.50	40.50
psbA	42.75	43.22	43.22	43.22	43.13	43.13	43.11	42.54

psbB	45.65	44.73	44.47	44.90	44.99	44.60	44.89	44.24
psbC	46.19	45.97	45.34	45.76	45.41	45.69	45.73	44.85
psbD	43.60	43.79	43.41	43.60	43.41	43.60	43.57	43.30
psbE	43.25	42.06	42.86	42.06	42.06	42.06	42.39	42.12
psbF	43.33	42.50	41.67	42.50	43.33	43.33	42.78	40.44
psbH	42.34	42.34	44.14	43.24	42.34	41.89	42.72	40.03
psbI	38.74	40.54	39.64	39.64	37.84	38.74	39.19	38.92
psbJ	43.90	44.72	45.53	45.53	42.28	43.09	44.17	43.04
psbK	38.17	38.71	38.71	39.78	38.71	39.78	38.98	37.77
psbL	30.77	30.77	30.77	30.77	31.62	31.62	31.05	31.02
psbM	30.48	29.52	29.52	29.52	30.48	30.48	30.00	30.92
psbN	47.73	46.21	47.73	46.97	46.21	46.97	46.97	45.56
psbT	35.09	36.84	35.09	35.96	33.33	35.09	35.23	36.54
psbZ	34.92	35.98	36.51	34.92	36.51	36.51	35.89	33.65
rbcL	44.82	44.40	44.26	44.33	44.19	43.91	44.32	44.32
rpl14	39.02	38.48	39.57	39.30	39.84	39.02	39.21	39.11
rpl16	45.59	46.57	45.59	45.59	45.34	45.83	45.75	43.69
rpl2	46.42	46.86	47.05	47.10	47.22	46.74	46.90	44.69
rpl20	38.26	38.82	38.28	38.48	37.15	37.57	38.09	36.98
rpl22	37.73	34.58	38.85	39.35	39.29	37.68	37.91	37.36
rpl23	37.62	40.29	39.93	42.16	40.40	42.86	40.54	37.96
rpl32	34.89	40.29	34.38	35.25	35.53	36.80	36.19	33.67
rpl33	39.04	38.31	40.30	37.31	37.81	37.81	38.43	37.12
rpl36	41.23	41.23	41.23	42.11	42.98	42.11	41.81	40.64
rpoA	37.58	37.61	37.97	39.56	38.42	39.10	38.37	36.09
rpoB	41.10	41.10	40.73	41.54	41.68	41.37	41.25	39.86

rpoC1	41.12	40.38	40.71	40.12	40.46	40.80	40.60	39.19
rpoC2	40.50	40.46	40.59	41.25	41.29	40.86	40.83	38.16
rps11	47.46	47.11	47.33	47.86	48.44	48.20	47.73	45.94
rps12	42.20	42.74	43.55	43.55	43.28	43.28	43.10	41.81
rps14	44.55	43.73	45.21	44.55	43.56	44.55	44.36	42.02
rps16	37.50	40.16	38.96	40.31	45.21	45.35	41.25	37.62
rps18	35.90	36.44	36.24	36.52	39.84	32.50	36.24	35.12
rps19	34.74	35.13	36.56	35.13	35.84	35.84	35.54	37.51
rps2	39.66	40.23	40.37	41.07	40.79	40.51	40.44	39.07
rps3	36.12	35.22	36.97	35.37	35.81	35.81	35.88	35.62
rps4	39.62	38.81	40.20	40.26	40.16	40.06	39.85	39.14
rps7	41.67	41.45	41.24	42.31	42.74	42.09	41.92	40.50
rps8	40.30	37.81	39.80	39.55	40.05	39.55	39.51	37.99
rrn16	56.67	56.54	56.54	56.47	56.30	56.37	56.48	56.62
rrn23	54.96	54.69	55.29	55.41	55.35	55.54	55.21	55.19
rrn4	52.43	51.46	52.43	52.43	52.43	52.43	52.27	50.10
rrn5	52.07	52.07	52.07	52.07	52.07	52.07	52.07	52.07
trnA-UGC	56.16	56.16	56.16	56.16	56.16	56.16	56.16	56.35
trnC-GCA	59.15	59.15	59.15	59.15	59.15	59.15	59.15	61.35
trnD-GUC	62.16	63.51	63.51	63.51	63.51	63.51	63.29	62.88
trnE-UUC	58.90	58.90	58.90	58.90	57.53	58.90	58.68	58.84
trnF-GAA	52.05	52.05	52.05	52.05	52.05	52.05	52.05	50.68
trnfM-CAU	56.76	56.76	56.76	55.41	54.05	55.41	55.86	57.03
trnG-GCC	53.52	52.11	52.11	53.52	53.52	53.52	53.05	52.11
trnG-UCC	53.52	53.52	53.52	53.52	53.52	53.52	53.52	53.52
trnH-GUG	57.33	57.33	57.33	57.33	57.33	57.33	57.33	57.69

trnI-CAU	45.95	45.95	45.95	45.95	45.95	45.95	45.95	46.31
trnI-GAU	59.72	59.72	59.72	59.72	59.72	59.72	59.72	59.72
trnK-UUU	54.17	55.56	55.56	55.56	56.94	55.56	55.56	55.23
trnL-CAA	50.62	50.62	50.62	50.62	50.62	50.62	50.62	50.53
trnL-UAA	47.06	48.24	47.06	48.24	48.24	48.24	47.84	48.16
trnL-UAG	57.50	58.75	57.50	56.25	57.50	56.25	57.29	57.17
trnM-CAU	42.47	39.73	41.10	41.10	41.10	41.10	41.10	42.28
trnN-GUU	52.78	52.78	52.78	52.78	52.78	52.78	52.78	52.87
trnP-UGG	48.65	47.30	47.30	48.65	50.00	48.65	48.42	48.47
trnQ-UUG	59.72	62.50	59.72	61.11	56.94	58.33	59.72	63.06
trnR-ACG	63.51	63.51	63.51	63.51	63.51	63.51	63.51	62.16
trnR-UCU	44.44	44.44	44.44	44.44	44.44	44.44	44.44	43.15
trnS-GCU	54.55	53.41	52.27	52.27	52.27	52.27	52.84	51.21
trnS-GGA	52.87	54.02	56.32	54.95	56.32	56.32	55.13	51.68
trnS-UGA	50.54	50.54	50.54	50.54	50.54	50.54	50.54	49.61
trnT-GGU	48.61	51.39	50.00	51.39	51.39	51.39	50.69	49.72
trnT-UGU	53.42	53.42	53.42	54.79	53.42	53.42	53.65	52.97
trnV-GAC	50.00	50.00	50.00	48.61	50.00	50.00	49.77	48.89
trnW-CCA	54.05	54.05	54.05	54.05	54.05	54.05	54.05	51.62
trnY-GUA	54.76	54.76	54.76	53.57	54.76	54.76	54.56	54.09
ycf1	34.68	35.01	36.05	35.77	35.75	36.21	35.58	32.76
ycf2	40.02	40.06	40.26	41.47	41.32	40.76	40.65	38.16
ycf3	39.38	39.38	39.45	39.77	38.99	39.77	39.45	39.41
ycf4	41.08	39.46	40.72	40.90	41.26	41.80	40.87	40.53

Supplementary Table S3. Optimal partitioning scheme, best fit substitution models, and related parameters for BI analysis. The sign after a gene, "-1", "-2", and "-3" indicate the nucleotide which is corresponding to the first, second or third position of the codon, respectively.

Subset	Partition names	Best Model	AICc	Sites (no.)
1	*rpl2*-2, *atpA*-1	GTR+I+G	4464.332	784
2	*atpE*-2, *atpA*-2, *petA*-2	GTR+I+G	4932.695	975
3	*petA*-3, *atpA*-3	GTR+G	11700.399	834
4	*rpl2*-1, *rps7*-1, *atpB*-1, *rpl14*-1	GTR+I+G	5670.7	1054
5	*rbcL*-2, *atpB*-2	GTR+I+G	4077.26	975
6	*petB*-3, *atpB*-3, *psaA*-3, *psbC*-3, *rbcL*-3, *atpI*-3	GTR+G	32280.225	2660
7	*rps8*-1, *rpl16*-1, *atpE*-1	GTR+G	2743.9434	411
8	*petD*-3, *rpl14*-3, *atpE*-3, *rpl16*-3, *rps19*-3, *rps3*-3, *ccsA*-3	GTR+G	17154.422	1244
9	*psbH*-1, *atpF*-1, *rpoC2*-1	GTR+G	13413.36	1661
10	*rps19*-2, *rps3*-2, *atpF*-2	GTR+G	3912.4072	520
11	*rpl33*-3, *rps8*-3, *rpl20*-3, *ycf4*-3, *atpF*-3, *cemA*-3, *rpoC2*-3	GTR+G	31060.957	2369
12	*atpH*-1, *petD*-1, *psbM*-1	GTR+I	1352.8522	290
13	*petB*-2, *psbD*-2, *psbF*-2, *atpH*-2	GTR+I+G	2052.3567	688
14	*rpoB*-3, *psaI*-3, *rpoC1*-3, *rps2*-3, *atpH*-3, *rps4*-3	GTR+G	27602.207	2334
15	*psaB*-1, *psaA*-1, *atpI*-1, *psbN*-1, *psbC*-1, *psbB*-1	GTR+I	12600.024	2756
16	*psbT*-2, *psbK*-2, *psbN*-2, *psaJ*-2, *atpI*-2	HKY+I	1787.7607	436
17	*rpl20*-1, *rpl32*-1, *psbF*-3, *ccsA*-1	GTR+I+G	5434.2676	577
18	*ccsA*-2	GTR+G	2660.7495	336
19	*rpl2*-3, *cemA*-1	GTR+G	4599.26	513
20	*cemA*-2	GTR+G	1702.9006	236
21	*matK*-1	GTR+G	6344.624	523
22	*rpoA*-2, *matK*-2	GTR+G	8192.79	886

23	*matK*-3	GTR+G	8029.8545	523
24	*petA*-1, *rpoC1*-1, *psbT*-1, *rps2*-1	GTR+G	8791.247	1308
25	*psbD*-1, *petG*-1, *psbE*-1, *petB*-1, *psbA*-1	GTR+I	3909.4453	1041
26	*psbL*-2, *petD*-2, *rpl14*-2	GTR+I	1277.2797	337
27	*petG*-2, *psbI*-2	HKY	217.60283	73
28	*ycf3*-3, *rps18*-3, *petG*-3, *psbH*-3	GTR+G	3797.1814	361
29	*rpl33*-2, *rpl33*-1, *petL*-1, *rps19*-1	GTR+I	1694.4982	276
30	*psbZ*-2, *petL*-2	HKY+I	474.05185	93
31	*psaJ*-3, *psaB*-3, *psbJ*-3, *psbK*-3, *petL*-3	GTR+G	10578.16	914
32	*ycf3*-1, *petN*-1	F81	815.79376	200
33	*psbJ*-2, *petN*-2	HKY+I	255.17438	69
34	*psbM*-3, *petN*-3, *psaC*-3, *rpl36*-3	GTR+G	2074.7522	181
35	*psbL*-1, *psaB*-2, *psbA*-2, *psbC*-2, *psaA*-2	GTR+I	7690.1743	2350
36	*psaC*-2, *psaC*-1	JC+I	451.21237	162
37	*rpoC2*-2, *psaI*-1, *psbK*-1	GTR+I+G	11664.144	1500
38	*psaI*-2, *psbM*-2	F81+I	265.36362	70
39	*psbJ*-1, *ycf4*-1, *rps8*-2, *psaJ*-1	GTR+G	2863.7822	405
40	*psbA*-3	GTR+G	3948.625	353
41	*psbB*-2	GTR+I	1816.7485	509
42	*psbB*-3	GTR+G	6766.048	509
43	*psbN*-3, *psbE*-3, *psbD*-3	GTR+G	4865.5737	479
44	*rps7*-2, *psbE*-2, *psbF*-1, *rps12*-2	SYM+G	1746.4741	405
45	*psbH*-2	K80+I+G	482.7741	75
46	*rrn5*, *psbI*-1	JC	492.08395	157
47	*rps16*-2, *psbI*-3, *rpl20*-2	HKY+G	2661.127	284
48	*psbZ*-3, *psbL*-3, *rpl23*-3	GTR	1760.9296	204

49	*psbT*-3, *rps14*-3	GTR+G	1737.0378	145
50	*rpl36*-2, *psbZ*-1, *ycf4*-2, *rps18*-2	HKY+G	2177.9822	361
51	*rrn4*, *rbcL*-1	GTR+I+G	2859.1157	578
52	*rpl16*-2	K80+G	784.3965	135
53	*rps3*-1, *rpl22*-1, *rps11*-1	GTR+G	4950.5215	521
54	*rpl22*-2, *rps16*-1	F81+I	2168.3906	218
55	*rpoA*-3, *rps11*-3, *rps16*-3, *rpl22*-3	GTR+G	11233.614	739
56	*ycf2*-1, *rpl23*-1, *rps14*-1, *rps4*-1, *rps18*-1	GTR+G	21025.404	2964
57	*ycf2*-2, *rpl23*-2	GTR+G	17389.873	2579
58	*rpl32*-2	SYM+G	489.81564	49
59	*rpl32*-3, *ycf1*-3	GTR+I+G	36315.047	2489
60	*rps12*-1, *rpl36*-1	F81+I	558.55743	165
61	*rpoA*-1	GTR+G	3903.0564	363
62	*rps14*-2, *rpoB*-1	GTR+G	6855.051	1182
63	*rps4*-2, *rpoB*-2, *rps2*-2, *rpoC1*-2	GTR+I+G	12479.668	2217
64	*rps11*-2	GTR+G	1248.0538	158
65	*rps12*-3, *rps7*-3	GTR+G	1651.6182	283
66	*rrn23*, *rrn16*	GTR+I+G	16627.736	4453
67	*ycf1*-1	GTR+I+G	31571.535	2440
68	*ycf1*-2	GTR+G	28205.08	2440
69	*ycf2*-3	GTR+G	18094.822	2477
70	*ycf3*-2	GTR+I	711.7614	171

Supplementary Table S4. Optimal partitioning scheme and related parameters using GTR+I+G substitution model for ML analysis. The sign after a gene, "-1", "-2", or "-3" indicate the nucleotide which is corresponding to the first, second or third position of the codon, respectively.

**Subset**	**Partition names**	**Model**	**AICc**	**Sites (no.)**
1	*atpA*-1, *atpB*-1, *rps7*-1	GTR+I+G	6268.2876	1162
2	*ycf3*-2, *atpA*-2, *atpB*-2	GTR+I+G	5378.0684	1178
3	*petB*-3, *atpB*-3, *petA*-3, *atpA*-3	GTR+I+G	21048.6	1549
4	*rpl16*-1, *atpE*-1, *psbT*-1, *rps2*-1, *rpoC1*-1, *petA*-1	GTR+I+G	10707.61	1584
5	*atpE*-2, *petA*-2	GTR+I+G	2528.9355	468
6	*rps3*-3, *rps19*-3, *rpl16*-3, *ccsA*-3, *petD*-3, *atpE*-3, *rpl14*-3	GTR+I+G	17156.523	1244
7	*rpoC2*-1, *atpF*-1, *psbH*-1	GTR+I+G	13414.986	1661
8	*atpF*-2, *rps3*-2	GTR+I+G	3167.943	425
9	*rps18*-3, *ycf3*-3, *atpF*-3, *psbH*-3, *petG*-3	GTR+I+G	6123.4604	546
10	*psbD*-1, *psbA*-1, *atpH*-1	GTR+I+G	2855.6096	787
11	*psbD*-2, *petB*-2, *psbJ*-2, *atpH*-2, *psbF*-2	GTR+I+G	2225.0498	728
12	*atpH*-3, *rps4*-3, *rpoB*-3, *rpoC1*-3, *rps2*-3, *psaI*-3	GTR+I+G	27604.215	2334
13	*psbB*-1, *atpI*-1, *ycf3*-1, *psaA*-1	GTR+I+G	7906.448	1677
14	*petN*-2, *psbI*-2, *petL*-2, *psaJ*-2, *psbZ*-2, *atpI*-2, *psbK*-2, *psbT*-2	GTR+I+G	2329.5837	551
15	*psbE*-3, *psbD*-3, *psaA*-3, *psbC*-3, *atpI*-3, *rbcL*-3	GTR+I+G	27400.133	2381
16	*rpl32*-1, *rpl20*-1, *psbF*-3, *ccsA*-1	GTR+I+G	5434.2676	577
17	*ccsA*-2	GTR+I+G	2661.7727	336
18	*rpl2*-3, *cemA*-1	GTR+I+G	4601.339	513
19	*cemA*-2, *matK*-2	GTR+I+G	6607.0225	759
20	*psbI*-3, *cemA*-3	GTR+I+G	3691.446	272
21	*matK*-1	GTR+I+G	6346.7783	523
22	*matK*-3	GTR+I+G	8031.9414	523
23	*psaB*-1, *psbC*-1, *psbN*-1, *petB*-1, *psbE*-1	GTR+I+G	6636.7085	1548
24	*psbM*-1, *petG*-1, *petD*-1	GTR+I+G	1166.0452	246

25	*petG*-2, *psbL*-1, *psbL*-2, *rpl14*-2, *petD*-2	GTR+I+G	1489.2417	414
26	*rpl33*-2, *rpl33*-1, *petL*-1, *rps19*-1	GTR+I+G	1696.685	276
27	*rpl36*-3, *psbN*-3, *petN*-3, *psaC*-3, *psbM*-3, *psbJ*-3, *petL*-3	GTR+I+G	3253.9524	295
28	*petN*-1, *psbE*-2, *psbF*-1, *rps12*-2, *rps7*-2, *rpl36*-1	GTR+I+G	2007.0156	471
29	*psaA*-2, *psbN*-2, *psbC*-2, *psaB*-2, *psbA*-2	GTR+I+G	7733.0425	2353
30	*psaJ*-3, *psaB*-3, *ycf4*-3, *psbK*-3	GTR+I+G	12073.361	1027
31	*psaC*-1, *psaC*-2, *psbI*-1, *rrn5*	GTR+I+G	956.1517	319
32	*psaI*-1, *ycf4*-1, *psaJ*-1, *rps8*-2, *psbJ*-1	GTR+I+G	3230.579	441
33	*psaI*-2, *psbM*-2	GTR+I+G	275.93295	70
34	*psbA*-3	GTR+I+G	3950.3474	353
35	*psbB*-2	GTR+I+G	1818.1273	509
36	*psbB*-3	GTR+I+G	6768.1284	509
37	*psbH*-2, *rpl36*-2, *rpl16*-2	GTR+I+G	1484.3793	247
38	*rpoC2*-2, *psbK*-1	GTR+I+G	11298.323	1464
39	*psbZ*-3, *rpl23*-3, *psbL*-3	GTR+I+G	1764.3126	204
40	*rps14*-3, *psbT*-3	GTR+I+G	1739.3445	145
41	*ycf4*-2, *psbZ*-1, *rps18*-2	GTR+I+G	1973.097	324
42	*rrn4*, *rbcL*-1	GTR+I+G	2859.133	578
43	*rbcL*-2	GTR+I+G	1818.5974	475
44	*rps8*-1, *rpl2*-1, *rpl14*-1	GTR+I+G	3139.1985	534
45	*rpoB*-1, *rps14*-2, *rpl2*-2	GTR+I+G	8409.454	1459
46	*rps16*-2, *rpl33*-3, *rpl20*-2	GTR+I+G	3123.2283	323
47	*rpoC2*-3, *rpl20*-3, *rps8*-3	GTR+I+G	22325.877	1689
48	*rps3*-1, *rps16*-1, *rpl22*-1, *rps11*-1	GTR+I+G	5895.759	616
49	*rpoA*-2, *rpl22*-2	GTR+I+G	4519.5938	486
50	*rpoA*-3, *rps11*-3, *rps16*-3, *rpl22*-3	GTR+I+G	11235.673	739

51	*ycf2*-1, *rpl23*-1, *rps18*-1, *rps14*-1, *rps4*-1	GTR+I+G	21028.19	2964
52	*ycf2*-2, *rpl23*-2	GTR+I+G	17391.18	2579
53	*rpl32*-2, *rps19*-2	GTR+I+G	1239.3536	144
54	*ycf1*-3, *rpl32*-3	GTR+I+G	36315.047	2489
55	*rpoA*-1	GTR+I+G	3905.1724	363
56	*rpoC1*-2, *rps2*-2, *rpoB*-2, *rps4*-2	GTR+I+G	12479.668	2217
57	*rps11*-2	GTR+I+G	1250.304	158
58	*rps12*-1, *rrn23*, *rrn16*	GTR+I+G	17060.018	4581
59	*rps7*-3, *rps12*-3	GTR+I+G	1653.5089	283
60	*ycf1*-1	GTR+I+G	31571.535	2440
61	*ycf1*-2	GTR+I+G	28205.672	2440
62	*ycf2*-3	GTR+I+G	18096.84	2477
